# Conservation physiology and the COVID-19 pandemic

**DOI:** 10.1093/conphys/coaa139

**Published:** 2021-01-12

**Authors:** Steven J Cooke, Rebecca L Cramp, Christine L Madliger, Jordanna N Bergman, Connor Reeve, Jodie L Rummer, Kevin R Hultine, Andrea Fuller, Susannah S French, Craig E Franklin

**Affiliations:** Fish Ecology and Conservation Physiology Laboratory, Department of Biology and Institute of Environmental and Interdisciplinary Science, Carleton University, Ottawa, Ontario K1S 5B6, Canada; School of Biological Sciences, The University of Queensland, Brisbane, Queensland 4072, Australia; Fish Ecology and Conservation Physiology Laboratory, Department of Biology and Institute of Environmental and Interdisciplinary Science, Carleton University, Ottawa, Ontario K1S 5B6, Canada; Fish Ecology and Conservation Physiology Laboratory, Department of Biology and Institute of Environmental and Interdisciplinary Science, Carleton University, Ottawa, Ontario K1S 5B6, Canada; Fish Ecology and Conservation Physiology Laboratory, Department of Biology and Institute of Environmental and Interdisciplinary Science, Carleton University, Ottawa, Ontario K1S 5B6, Canada; ARC Centre of Excellence for Coral Reef Studies and College of Science and Engineering, James Cook University, Townsville, Queensland 4811, Australia; Department of Research, Conservation and Collections, Desert Botanical Garden, Phoenix, AZ 85008, USA; Brain Function Research Group, Department of Research, Conservation and Collections, School of Physiology, Faculty of Health Sciences, University of the Witwatersrand, Parktown 2193, South Africa; The Department of Biology and The Ecology Center, Utah State University, Logan, UT 84322, USA; School of Biological Sciences, The University of Queensland, Brisbane, Queensland 4072, Australia

**Keywords:** Coronavirus, environmental change, lockdown, pandemic, wildlife, zoonoses

## Abstract

The COVID-19 pandemic and associated public health measures have had unanticipated effects on ecosystems and biodiversity. Conservation physiology and its mechanistic underpinnings are well positioned to generate robust data to inform the extent to which the Anthropause has benefited biodiversity through alterations in disturbance-, pollution- and climate change-related emissions. The conservation physiology toolbox includes sensitive biomarkers and tools that can be used both retroactively (e.g. to reconstruct stress in wildlife before, during and after lockdown measures) and proactively (e.g. future viral waves) to understand the physiological consequences of the pandemic. The pandemic has also created new risks to ecosystems and biodiversity through extensive use of various antimicrobial products (e.g. hand cleansers, sprays) and plastic medical waste. Conservation physiology can be used to identify regulatory thresholds for those products. Moreover, given that COVID-19 is zoonotic, there is also opportunity for conservation physiologists to work closely with experts in conservation medicine and human health on strategies that will reduce the likelihood of future pandemics (e.g. what conditions enable disease development and pathogen transfer) while embracing the One Health concept. The conservation physiology community has also been impacted directly by COVID-19 with interruptions in research, training and networking (e.g. conferences). Because this is a nascent discipline, it will be particularly important to support early career researchers and ensure that there are recruitment pathways for the next generation of conservation physiologists while creating a diverse and inclusive community. We remain hopeful for the future and in particular the ability of the conservation physiology community to deliver relevant, solutions-oriented science to guide decision makers particularly during the important post-COVID transition and economic recovery.

## Teaser

The COVID-19 pandemic has had unanticipated effects on ecosystems and biodiversity. The conservation physiology toolbox can be used to characterize the Anthropause, mitigate the zoonotij aayc outbreaks and address the emerging issues. Conservation physiology is well positioned to deliver solutions-oriented science to guide decision makers particularly during the important post-COVID transition and economic recovery.

## The COVID-19 pandemic

At the end of 2019, few people had heard the acronym COVID-19 or could imagine the devastating and long-lasting toll that severe acute respiratory system coronavirus 2 would have on humanity. From loss of life [World Health Organization (WHO), 2020], economic crises (Fernandes, 2020), degradation of human well-being (Fore, 2020) and food insecurity (Laborde *et al.*, 2020), the pandemic and various public health responses will long be remembered as a defining event in human history (Morens *et al.*, 2020). Current public health and medical efforts are focused towards the use of evidence-based public health measures to slow the spread of the virus, encourage vaccine development and improve therapeutics and other medical interventions to support infected individuals. There are also ongoing discussions about what the global response to the pandemic means for biodiversity, environment and our relationship with the natural world (e.g. Corlett *et al.*, 2020; Shakil *et al.*, 2020).

The pandemic has led to major changes in human activity related to public health measures. At one point (in spring of 2020), more than half of the human population was sheltering in place with dramatic reductions in mobility. These restrictions have continued in various forms in most jurisdictions to address subsequent waves or most recently over concern for viral variants. Some have labelled this period as the ‘Anthropause’, recognizing that it is a unique opportunity to understand how human activity has impacted biodiversity (Rutz *et al.*, 2020). Also salient is that COVID-19 is a zoonotic disease (Ahmad *et al.*, 2020; Chakraborty *et al.*, 2020) believed to have infected humans via consumption of wildlife (likely bats, but there may have been another intermediate mammalian host; Malik *et al.*, 2020). Although the pandemic arose from human–wildlife interactions, the response to the pandemic has meant that human impacts on biodiversity and the environment may be muted (Zambrano-Monserate *et al.*, 2020). However, there are instances in which the response to the pandemic has had (or will have) negative impacts on biodiversity and the environment (e.g. diverting attention from climate change, loosening of environmental regulations or curtailed enforcement, loss of funding from ecotourism for conservation, reduced wildlife protection or poaching enforcement, impairing current on-the-ground conservation and recovery activities; Corlett *et al.*, 2020; Zambrano-Monserate *et al.*, 2020). Moreover, it is anticipated that post-pandemic economic stimulus measures could further threaten biodiversity loss unless the transition is used as an opportunity to achieve a ‘greener’ future (McElwee *et al.*, 2020; Sandbrook *et al.*, 2020). Even though the health and wellness of humans are top of mind, given the inherent connections between human well-being and the environment (Dasgupta, 2001), the environment and biodiversity are also of great importance and are reflected in a number of the United Nations Sustainable Goals. In fact, the ‘One Health’ concept (i.e. a collaborative, multi-sectoral and trans-disciplinary approach—working at local, regional, national and global levels—to achieve optimal health and well-being outcomes recognizing the interconnections between people, animals, plants and their shared environment) has become a logical way to frame environmental issues related to COVID-19 (Bonilla-Aldana *et al.*, 2020).

Conservation physiology involves the use of physiological tools, knowledge and concepts to understand and solve conservation problems (Cooke *et al.*, 2013; Madliger *et al.*, 2021). While being one of the many subdisciplines of conservation science (Kareiva and Marvier, 2012), particular strengths of conservation physiology include understanding mechanisms and unravelling complex interactions to inform policy and practice (Cooke and O’Connor, 2010). As conservation scientists engage in science to understand the Anthropause and address the issue of human–wildlife interactions in the context of zoonoses, there are obvious ways in which the conservation physiology community can play an active and important role. Here, we briefly reflect on the ways in which conservation physiology has, or could play, a meaningful role in characterizing the Anthropause, enabling post-COVID-19 recovery and transitions in a manner that benefits biodiversity and humans, and in understanding and mitigating zoonoses. The pandemic has also impacted the conservation physiology community and practitioners in diverse ways (e.g. reductions in funding, research interruptions), and so we conclude by reflecting on those impacts and the ways in which our community can continue to support one another and create an inclusive and caring environment. Although our focus is on conservation physiology, we acknowledge that many of the ideas and issues explored here are germane to the broader realm of conservation science.

## Conservation physiology for the Anthropause and beyond

The Anthropause is an unprecedented opportunity to use a ‘natural experiment’ to understand the effects of humans on biodiversity and the environment (Bates *et al.*, 2020). In general, it is assumed that lockdowns have restricted human movement and activity and thus reduced direct interactions with nature to the benefit of biodiversity (Corlett *et al.*, 2020) as well as reduced pollution (Muhammad *et al.*, 2020) and greenhouse gas emissions (Dutheil *et al.*, 2020; Eroğlu, 2020). In reality, the situation has been more complex. For example, although pollution was reduced early on due to the temporary closure of industries, there is a concern that environmental regulations will be relaxed to enable rapid economic recovery (Helm, 2020). Similarly, although human outdoor activity was reduced initially, connections with nature became important for mental health (especially for people living in urban centres; Venter *et al.*, 2020) while the food insecure have had to look to natural areas for food (Béné, 2020; Pinder *et al.*, 2020). A full discussion of these topics is beyond the scope of this paper.

Here, we briefly consider how conservation physiology and the conservation physiology toolbox (see Madliger *et al.*, 2018) have been, or could be, used to inform our understanding of the Anthropause on different biodiversity and environmental threats. We recognize that the pandemic and public health response impeded the ability of researchers to conduct research in some regions such that the Anthropause (especially the earliest examples during lockdowns in February through April of 2020) will never be studied in a complete or systematic way. Yet, the Anthropause is still ongoing or being repeated in some areas due to seasonal waves, thus allowing for proactive research planning. Moreover, physiological indicators are sensitive (Madliger *et al.*, 2018); therefore, even if used *post hoc*, they may provide important insight into what wildlife experienced during the Anthropause. Some physiological tools (e.g. biologging and biotelemetry) can be used remotely such that there may be good examples of time series that extend throughout the entirety of the period (Rutz *et al.*, 2020). It is unlikely for most species that change in population size would be evident so quickly, but physiology could tell us what we might expect if the pause was longer or if we work together to achieve sustainable or ongoing Anthropause-like reductions in human impacts on biodiversity and the environment.

### Pollution

Conservation physiology is well equipped to assess the ways in which various levels of different pollutants have impacted wildlife and ecosystems during the Anthropause. In general, it is believed that water and air pollution decreased at least initially during the early phases of lockdown. We anticipate that these reductions may have been sufficient to be reflected in wildlife and ecosystem health. Use of health-related biomarkers would be particularly relevant for assessing the effects of the Anthropause on wildlife (Galloway, 2006; Luna-Acosta *et al.*, 2010). For example, some preliminary research suggests that reductions in noise pollution has altered bird songs and reduced stress in passerines (Kleist *et al.*, 2018; Derryberry *et al.*, 2020). In a post-COVID-19 context, there is fear that environmental regulations will be diluted or not enforced in an effort to enable economic recovery. Conservation physiology can be used to identify instances where changes to the type or severity of pollution are influencing wildlife (either negatively or positively) and to serve as an early warning system if pollution thresholds are having negative effects on wildlife.

### Environmental change

Reductions in greenhouse gas emissions (especially CO_2_) and other pollutants arising from industrial closures and reduced air and vehicle travel were among the first environmental changes to be widely documented during the pandemic (Boretti, 2020; Sinabell *et al.*, 2020). Global environmental change is a chronic problem with high levels of interannual variation (e.g. in temperature) such that it is unclear if the changes observed during the Anthropause will have measurable effects on biodiversity. However, the Anthropause did demonstrate what is possible. If economic recovery efforts favour initiatives and incentives that enable progress on climate change (see Hepburn *et al.*, 2020), there is a possibility for a widespread, long-term benefit. Time series data on plant and animal physiological states will be important for assessing the effects of these actions on ecosystems. For example, documenting how roadside plant communities respond to reduced vehicle traffic and subsequent reductions in ozone and nitrogen deposition exposure through time could yield valuable information (Kenkel *et al.*, 2016; Feng *et al.*, 2019). Moreover, it is more important than ever to conduct robust studies on environmental change to ensure that greenhouse gas reduction targets are meaningful and benefit all biodiversity.

### Human disturbance

Human activities (ranging from hiking to motorboat traffic) can disturb ecosystems and fauna, inducing stress to levels that cause population-level declines (Wikelski and Cooke, 2006; see Müllner *et al.*, 2004, for example). While sheltered in place, human disturbance was presumably reduced as evidenced by anecdotal reports of wildlife roaming empty streets in urban areas (Singh, 2020). There are also a growing number of such examples in the peer-reviewed literature spanning reductions in wildlife collisions and expansion of breeding habitats (Manenti *et al.*, 2020; Zellmer *et al.*, 2020). Reductions in human disturbance may be even more extreme in remote locations. For example, normally around 40 000 visitors descend on Antarctica in a given year for ecotourism; this year, that number is predicted to be less than 1000 (Quinn, 2020). In the Caribbean, some scientists have predicted that reductions in food provisioning of iguanas by ecotourists could demonstrate the negative consequences of that activity (French, 2020; Stokstad, 2020). For example, changes in food provisioning may impact oxidative stress, energy metabolites, gut microbiome and body condition (French, 2020). Physiological biomarkers such as glucocorticoids, reproductive hormones and oxidative stress metrics (Madliger and Love, 2014; French *et al.*, 2017) or electronic tagging tools that incorporate physiological sensors (Cooke *et al.*, 2004) are particularly powerful for assessing disturbance in wildlife. In fact, the International Biologging Society has launched a COVID-19 biologging initiative in an attempt to study the Anthropause (Rutz *et al.*, 2020). Some stress biomarkers in certain tissues [e.g. glucocorticoid profiles in baleen (Fernández Ajó *et al.*, 2018), hair (Koren *et al.*, 2019) and feathers (Freeman and Newman, 2018)] can be used to retroactively reconstruct stress or reproductive states over periods of months or years, which creates unique opportunities to study the Anthropause.

### Invasive species

The pandemic reduced global trade and human movement such that there were presumably reductions in the movement and introduction of invasive species. It is difficult to know what would have been introduced if the pandemic did not occur. However, biosecurity in the form of invasive species prevention and control efforts were presumably impacted in some regions (see Kerr, 2020). The application of physiological tools could help to refine control efforts to improve success and compensate for reductions in control during the pandemic (e.g. Lennox *et al.*, 2015; see Siefkes, 2017, for example). This is particularly salient for recent invasions where rapid control efforts could be successful. Similarly, physiological tools could be used to assess the effects of invasive species on endemic communities, especially where control efforts were reduced. There is also much that could be learned from efforts to monitor the spread of COVID-19 and thinking about it as being analogous to an invasive species (Bertelsmeier and Ollier, 2020).

### Emerging COVID-19 threats

The response of human civilization to COVID-19 has certainly led to changes that define the Anthropause (summarized above), yet the pandemic has also led to several emerging threats. Most notably has been the widespread use of antimicrobial cleansers (e.g. hand sanitizers, wipes, sprays) used to clean hands and high-touch surfaces (e.g. door handles, grocery cart handles) or to fumigate large indoor and outdoor spaces (Klemeš *et al.*, 2020). Indeed, disinfectant demand is extremely high and established producers are unable to meet the demand (Terlep and Sebastian, 2020). Although an important aspect of public health (Xiao and Torok, 2020), there is an emerging concern regarding the effects of these disinfectants on wildlife (especially in urban areas; Nabi *et al.*, 2020). Excessive use of these substances could lead to introductions to aquatic systems via stormwater runoff or through sanitary sewage systems. Chlorine, which is a powerful biocide with manifold negative effects on aquatic ecosystems, is being widely used as a disinfectant causing much concern (García-Ávila *et al.*, 2020). Disinfectants may also impact terrestrial organisms such as some birds, insects and mammals through direct exposure when spayed liberally from vehicle-based or backpack diffusers (Palmer and Barth, 2020). There are anecdotal reports of animal mortality attributed to the use of disinfectants (You, 2020), but there is a need for science to inform decision makers about the toxicity of these substances relative to different modes of deployment. There is also a concern that the frequent use of disinfectants may spark a rise in antimicrobial resistant superbugs that could result in a ‘slow-moving pandemic’ in the future (see Burrows, 2020). Beyond cleansers, plastic waste is being generated in the form of disposable masks (Ammendolia *et al.*, 2020; Roberts *et al.*, 2020; Silva *et al.*, 2020) as well as other consumer products (e.g. more single-use plastic cups rather than refilling and reusing them; Adyel, 2020). The conservation physiology community is well positioned to generate cause-and-effect relationships and identify regulatory limits to protect wildlife for both cleansers and plastic waste.

### Conservation physiology and zoonoses

Zoonoses have come to the forefront of public thinking in 2020, as it became apparent that COVID-19 was passed from wildlife to humans. Pandemics arising from human–wildlife interactions are not new (Santana, 2020), and for decades, there have been warnings raised about an emerging disease in wildlife and what it means for biodiversity and human health (Daszak *et al.*, 2000; Jones *et al.*, 2008). The combination of direct human–wildlife interactions with increasing environmental change means that, with near certainty, this type of event will become more common (Tollefson, 2020). Society is now acutely aware of the link between wild animal stress and viral shedding rates (Gibbens, 2020). As such, efforts to create environmental conditions (e.g. minimal disturbance, pollution, habitat alteration, etc.) that benefit the environment and wildlife will also benefit people (i.e. exactly what the One Health movement espouses; Bonilla-Aldana *et al.*, 2020; Gruetzmacher *et al.*, 2020). Indeed, UNESCO released a statement on 20 May 2020 that stated, ‘The appearance of COVID-19 has shown that when we destroy biodiversity we destroy the system that supports human life. The more biodiverse an ecosystem is, the more difficult it is for a pathogen to spread rapidly or dominate. Loss of biodiversity provides an opportunity for pathogens to pass between animals and people. We must learn and adapt faster than ever, and the COVID 19 virus has lessons that apply to global crises of biodiversity loss. For this reason, our best vaccine for the future is to protect nature and biodiversity. It is no longer just a matter of ecology but of being aware that if we want to reduce the occurrence of pandemics, we must have a healthy nature.’ This is a poignant reminder of the important role that can be played by conservation physiologists.

There have been calls for developing integrated surveillance-response systems to detect and prevent future pandemics with an important component of those efforts including wildlife health monitoring (Zinsstag *et al.*, 2020). There are real opportunities now to build capacity to monitor and manage potential pathogens using physiological markers at key human/wildlife interfaces to benefit both humans and wildlife. Conservation physiologists work closely with veterinary and animal health experts (i.e. conservation medicine; Ostfeld *et al.*, 2002; Daszak *et al.*, 2004), and there are already calls for closer collaboration among relevant disciplines (Decaro *et al.*, 2020). Given the need to better understand the specific mechanisms by which environmental change and other human-induced impacts on ecosystems and wildlife mediate disease transmission and pathogenicity, conservation physiologists have much to offer. Given the apparent role of bats in the COVID-19 pandemic, there is a need for specific research on environmental mediators of disease development in those taxa (Rocha *et al.*, 2020). There is also a need for experimental studies to determine the potential for reverse zoonotic transmission of COVID-19 (and other pathogens) to wildlife—a concerning but real possibility (Olival *et al.*, 2020). Finally, there are opportunities to piggyback off of the massive investment in COVID-19 vaccine development and technology for diseases of significance to wildlife, which would require more collaboration between the conservation medicine and conservation physiology communities.

**Figure 1:**
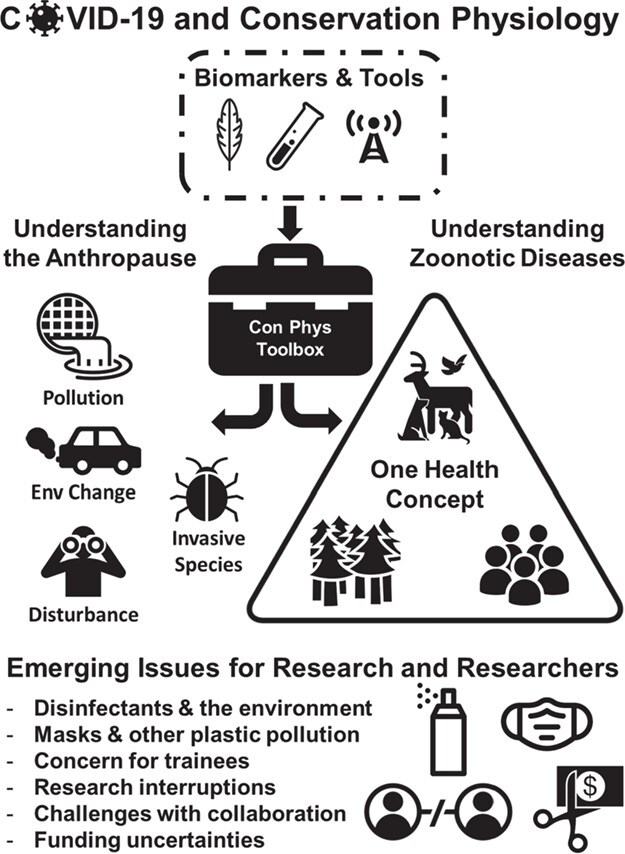
Infographic visualizing aspects of COVID-19 and conservation physiology. The conservation physiology toolbox includes various physiological biomarkers (e.g. glucocorticoids in feathers, oxidative stress indicators in blood) and electronic tag sensors to assess organismal responses to different challenges. The Anthropause has led to changes in pollution, environmental change, human disturbance and invasive species introductions and control, which can all be studied using the conservation physiology toolbox. Moreover, the toolbox can be used to study how human activities influence the environment, thus creating conditions in animals that favour emergence of zoonoses. COVID-19 has also created emerging issues that require conservation physiology research (e.g. effects of disinfectants and plastic medical waste on biodiversity), while at the same time impacting the conservation physiology research community.

## COVID-19 and the conservation physiology community

Conservation physiologists are, first and foremost, humans. It is therefore not surprising that COVID-19 has impacted the conservation physiology community in a number of ways. Some of these are rather obvious, such as impacts on health and wellness associated with the isolation and loneliness that have accompanied the pandemic. Others include changes in time available to devote to research, student training or writing, as scholars focus on self-care, family or important but altered work duties (e.g. online teaching). These impacts have not been felt uniformly across the community, with women being burdened more heavily than their male colleagues (Gabster *et al.*, 2020; Malisch *et al.*, 2020). At *Conservation Physiology*, we did see an increase in submissions (roughly a doubling) between May and September 2020, suggesting that the pandemic did provide some with the opportunity to write. At journals where author gender is tracked, there is evidence of fewer female-led contributions since COVID-19 (King and Frederickson, 2020). The long-term consequences of COVID-19-related interruptions on women in science are unknown but presumably erase recent gains (Gabster *et al.*, 2020). As a community, we need to support female scholars, particularly early career scientists who are just now gaining traction in their careers.

We also recognize that the pandemic has had differential effects with marginalized communities (including indigenous communities; see Curtice and Choo, 2020) and those from lower-income regions or communities being disproportionately impacted by the pandemic. Moreover, while the world suffered with COVID-19, racial injustice garnered attention with the Black Lives Matter movement creating calls for a meaningful action (Çetinkaya *et al.*, 2020; Gravlee, 2020). Racism and bias remain problems in the scientific community with much room for change (Nelson, 2019). Our editorial team has recently called for a more inclusive and diverse approach to conservation physiology (see Cooke *et al.*, 2020a), and we look forward to working with our community to make that happen.

The conservation physiology community, like others, is facing job losses, hiring freezes and salary cuts. A recent informal survey by the American Physiological Society (APS) (2020) reported that 28% of the respondents had experienced salary cuts or furloughs. These job-related changes cause significant anxiety, making it difficult to engage in research. Moreover, there are research funding cuts that have already occurred and are expected to continue for years to come. The same APS survey revealed that ~82% of the established researchers believe that the pandemic will negatively impact their ability to apply for grants and maintain continuous funding. These cuts also extend to unique facilities (e.g. research stations, zoos, botanical gardens). Field stations have lost revenue due to closures or reductions in use during the pandemic (see Kaiser, 2020) and donations are also down. In many cases, ecotourism operations help to fund conservation and research. Zoos, aquaria, natural history museums, wildlife parks and botanical gardens that depend partially on revenue generated from visitors have suffered greatly (Brulliard and Oldham, 2020). Much conservation physiology research is conducted by research staff at those institutions, but with loss of revenue, efforts are currently focused on core maintenance (e.g. animal husbandry). The long-term consequences of this transition are unclear but may impede collaboration and research and have direct consequences for conservation (Briggs, 2020). International collaboration has additionally been minimized with reductions in international travel. This is particularly concerning for regions with relatively little science capacity where collaboration with researchers from developed nations is vital for capacity building. The scientific community has quickly pivoted to internet-based video conferencing that does create the potential for longer-term collaboration and capacity building so there could be some promising developments.

The *Conservation Physiology* editorial team wishes to also note our concern for the next generation of conservation physiologists. For example, during the pandemic, youth are not being exposed to formal outdoor and environmental education (Quay *et al.*, 2020), which is regarded as an inspirational gateway to the conservation and environmental sciences (Morana *et al.*, 2012). Yet, there is evidence that during the lockdown, people are reconnecting with their own backyards (Carroll, 2020). There is uncertainty regarding the ability to recruit future conservation physiology trainees. In fact, the APS survey revealed that nearly half (49%) of the faculty respondents noted that they had to lay off skilled staff or had been unable to hire staff (including undergraduate students) due to the pandemic. For those early career researchers who have already decided to embark on a career in conservation physiology, many are facing interruptions in education and training. Laboratory classes and other in-person learning activities have been cancelled at many institutions in favour of online instruction (Paudel, 2020). Internships and other training experiences that embed learners within government laboratories or communities have been halted; field trips to other regions have been delayed or cancelled; ongoing field and laboratory work has been disrupted or halted altogether; and conferences, where early career researchers would normally share their work and network with others, have been postponed or moved online, which reduces valuable networking opportunities for young scientists. In fact, the APS survey revealed that 83% of the trainee respondents indicated that their conference plans were thwarted. There have also been hiring freezes, which limits job prospects for recent graduates. As a community, we need to support our early career researchers and recognize that the pandemic will have impacted their training in various and inconsistent ways. We will need to be understanding of these challenges when assessing performance for awards or hiring new team members. Most importantly, we need to take the time to listen to the next generation of conservation physiologists and do what we can to help them connect with the discipline in the same ways that made us excited about science.

The journal *Conservation Physiology* has continued its activities during the pandemic, with a focus on rigourous peer review but recognizing that referees and editorial team members are facing unique pressures. It has been more difficult to secure reviewers and sometimes it takes a little longer (e.g. to secure referees, for referees to do their work, and for editors to do their work), but we are grateful for those who have supported us during the pandemic. The 2-week review window was extended to 3 weeks with additional flexibility and understanding when editors nudge tardy reviewers and authors have been given longer time to revise their work. The editorial team has been working hard, but we have also faced challenges with keeping up with duties, so we thank authors for their patience during this time. The take-home message is that the journal continues to provide rigourous review and publish high-quality manuscripts thanks to the support of our community. We welcome any feedback on what the journal editorial team can do to make any aspects of our processes better and more responsive to the challenges that you—our community—are facing.

Lastly, it is necessary to recognize that COVID-19 can be lethal and we are aware of at least one conservation physiology scholar who died as a result of COVID-19-related complications—Dr Ron O’Dor from Dalhousie University (http://oceantrackingnetwork.org/in-memoriam-dr-ronald-odor/). Ron was known for his work on squid, where he focused on understanding their physiology and behaviour in an effort to ensure that these often-forgotten animals were recognized for their important roles in marine ecosystems. It is likely that there are others in our community that we have lost, and we wish to acknowledge the human toll that this pandemic has had.

## A hopeful future

As outlined above, conservation physiology has much to offer the study of the Anthropause. It is still early days, however, and it is necessary to mine existing data, be opportunistic and creative and remain optimistic. Failure to engage now and in the near future may impede the ability to achieve the necessary transition that is hoped for during post-COVID-19 recovery. Conservation physiology has the potential to elucidate cause-and-effect relationships and identify thresholds, both of which are essential for understanding how different stressors affect wild organisms and to enable evidence-based decision making (Kadykalo *et al.*, 2020). We anticipate an emergence of studies in the near future that use biologging and biotelemetry tools equipped with physiologically relevant sensors (e.g. acceleration, electrocardiogram), physiological biomarkers (e.g. glucocorticoids, oxidative stress indicators) and genomic tools to assess the condition of organisms before, during and after the Anthropause (Fig. 1). However, there is also a pressing need for science on the effects of different COVID-19-related antimicrobials, disinfectants and plastic waste on ecosystems, which is another obvious opportunity for the conservation physiology community. Given that COVID-19 is zoonotic, conservation physiology also has the potential to determine the conditions that enable disease development and pathogen transfer (Fig. 1). Finally, as the conservation physiology community adjusts to the reality of COVID-19, there is a need to ensure that we focus on supporting early career researchers and continue to create a diverse and inclusive community. Although 2020 has been a challenging year, we are hopeful for the future and in particular the ability of the conservation physiology community to deliver relevant, solutions-oriented science to guide decision makers (Cooke *et al.*, 2020b; Fig. 1). Conservation physiology remains highly relevant as the Anthropocene continues (Madliger *et al.*, 2017)—the Anthropause will presumably be short-lived, but what will matter will be what we can learn and how we can improve the protection, conservation and restoration of biodiversity.

## Funding

S.J.C. is supported by the Natural Sciences and Engineering Research Council of Canada.
